# Osteopontin in Pulmonary Hypertension

**DOI:** 10.3390/biomedicines11051385

**Published:** 2023-05-07

**Authors:** Argen Mamazhakypov, Abdirashit Maripov, Akpay S. Sarybaev, Ralph Theo Schermuly, Akylbek Sydykov

**Affiliations:** 1 Department of Internal Medicine, Excellence Cluster Cardio-Pulmonary Institute (CPI), Member of the German Center for Lung Research (DZL), Justus Liebig University of Giessen, 35392 Giessen, Germany; 2 Department of Mountain and Sleep Medicine and Pulmonary Hypertension, National Center of Cardiology and Internal Medicine, Bishkek 720040, Kyrgyzstan

**Keywords:** osteopontin, pulmonary hypertension, biomarkers, right heart failure

## Abstract

Pulmonary hypertension (PH) is a pathological condition with multifactorial etiology, which is characterized by elevated pulmonary arterial pressure and pulmonary vascular remodeling. The underlying pathogenetic mechanisms remain poorly understood. Accumulating clinical evidence suggests that circulating osteopontin may serve as a biomarker of PH progression, severity, and prognosis, as well as an indicator of maladaptive right ventricular remodeling and dysfunction. Moreover, preclinical studies in rodent models have implicated osteopontin in PH pathogenesis. Osteopontin modulates a plethora of cellular processes within the pulmonary vasculature, including cell proliferation, migration, apoptosis, extracellular matrix synthesis, and inflammation via binding to various receptors such as integrins and CD44. In this article, we provide a comprehensive overview of the current understanding of osteopontin regulation and its impact on pulmonary vascular remodeling, as well as consider research issues required for the development of therapeutics targeting osteopontin as a potential strategy for the management of PH.

## 1. Introduction

Pulmonary hypertension (PH) is a condition affecting pulmonary vasculature [1] and is hemodynamically defined as a mean pulmonary artery pressure (mPAP) greater than 20 mmHg at rest as assessed by right heart catheterization [2]. PH is classified into five main clinical groups: pulmonary arterial hypertension (PAH), PH resulting from left heart disease, PH resulting from chronic lung disease or hypoxia, chronic thromboembolic PH (CTEPH), and PH with unclear or multifactorial mechanisms [2]. PH represents a chronic and ultimately fatal pulmonary vascular disorder of multifactorial origin [3]. The primary pathological characteristics of PH include persistent pulmonary vascular constriction and excessive obstructive pulmonary vascular remodeling [4]. At the cellular level, initial pathological mechanisms of pulmonary vascular remodeling are characterized by dysfunction and apoptosis of pulmonary artery endothelial cells (PAECs). At later stages, hyperproliferation and apoptosis resistance of pulmonary artery smooth muscle cells (PASMCs) lead to structural changes in the pulmonary vasculature, elevation in pulmonary arterial pressure (PAP), and pulmonary vascular resistance, which ultimately culminate in right ventricular (RV) failure [5].

Despite the multitude of studies conducted in the field of PH research, the underlying mechanisms of this condition remain an unresolved issue. A plethora of pathological processes, such as an inherent genetic predisposition [6], aberrant metabolic processes [7,8], perturbations in apelin signaling pathways [9], aberrations in calcium signaling pathways [10], DNA damage [11], mitochondrial dysfunction [12], and dysregulation of micro RNAs (miRNAs) [13], has been implicated in the pathogenesis of PH. Various matricellular proteins, including osteopontin, have been identified as critical mediators in the pathogenesis of pulmonary vascular remodeling. Matricellular proteins, as a class of inducible, multifunctional, secretory, and non-structural proteins, can act as cytokines on a plethora of cellular mechanisms and function as extracellular matrix proteins located between cells [14]. In this article, we provide a comprehensive overview of the recent advancements in our understanding of the role of osteopontin in the pathogenesis of pulmonary vascular remodeling, gleaned from various experimental in vitro studies and animal models, and clinical studies. We will also delve into the challenges faced in the investigation of osteopontin in PH and explore its potential as a viable therapeutic target.

## 2. Osteopontin Signaling

The name “osteopontin”, which is composed of two words, was proposed by Oldberg and colleagues [15]. The prefix osteo- is derived from “osteon”, the Greek word for bone, and reflects the fact that osteopontin was first isolated from the mineralized bone matrix of bovines as a bone sialoprotein I [16]. The suffix -pontin is derived from “pons”, the Latin word for bridge, and reflects osteopontin’s role as a linking protein between cells and hydroxyapatite in the matrix [15]. Osteopontin is also known as secreted phosphoprotein 1, uropontin, and early T-lymphocyte activation-1.

As soon as osteopontin was isolated, it was revealed that the matricellular protein osteopontin is a cytokine that is synthesized and expressed by a wide array of cells and tissues in the body. The tissues and organs expressing osteopontin include kidney, inner ear, brain, heart, lung, vessels, skin, and bone marrow [17], as well as luminal epithelial surfaces of the gastrointestinal tract, gall bladder, pancreas, urinary and reproductive tracts, lung, breast, salivary glands, and sweat glands [18]. Osteopontin is also found in biological fluids, such as blood, milk, urine, and seminal fluid [17]. Osteopontin expression is considerably upregulated in response to a variety of pathological processes, including inflammation, mechanical stress, and tissue injury and repair. In various cardiovascular diseases, synthesis of osteopontin is induced in smooth muscle cells and cardiomyocytes [17].

Osteopontin plays a multifarious role in biological processes such as inflammation, immunological response, wound healing, cellular adhesion, migration, survival, and biomineralization. Binding of osteopontin to integrins activates signaling pathways that regulate diverse cellular functions including cell proliferation, adhesion, invasion, migration, and fibrosis [19]. 

Osteopontin also interacts with CD44, a ubiquitously expressed cell-surface receptor, which exists as a number of isoforms [20]. It is encoded by 20 exons, 10 of which are constant and form the invariant extracellular domain of the smallest standard isoform [21]. The variant isoforms are generated by alternative splicing and may contain in addition to 10 constant exons a single variant exon or a combination of variant exons [21]. CD44 has been shown to be involved in cell–matrix and cell–cell interactions [22]. Although hyaluronic acid is considered as a principal ligand for CD44, other extracellular-matrix proteins including serglycin, collagen, fibronectin, chondroitin sulfate, laminin, and osteopontin also may serve as ligands for CD44 [22]. Osteopontin does not interact with the standard isoform of CD44, but rather binds to its variant isoforms [23], such as CD44v6 and CD44v7 [24,25]. 

Variant CD44 isoforms play critical roles in the development of various cancers through their interaction with osteopontin [21]. The exact role of the osteopontin-mediated activation of CD44 variants in cardiovascular diseases remains poorly explored and the literature is scarce. It was demonstrated that the osteopontin–CD44v6 interaction mediates calcium deposition in valve interstitial cells from patients with noncalcified aortic valve sclerosis [26].

Histological studies revealed CD44 expression in lung plexiform lesions from patients with idiopathic PAH (IPAH) [27]. Another study showed that adventitial fibroblasts isolated from chronically hypoxic hypertensive calves displayed increased expression of CD44 along with αVβ3 and osteopontin [28]. However, these studies did not investigate which CD44 isoforms were upregulated, and did not demonstrate an interaction between CD44 and osteopontin. Recently, CD44v7–10, CD44v8–10, CD44v9–10, and CD44v10 transcripts were detected in lungs of mice subjected to chronic hypoxia [29]. In PAH patients, the CD44v8–10 variant was expressed by endothelial-to-mesenchymal transition-like PAECs in pulmonary vessels with neointimal hyperplasia or occluded vessels, including plexiform lesions [29]. It remains, however, to be elucidated whether osteopontin interacts with these CD44 variants and which signaling pathways are activated in the setting of PH.

Recent studies showed that osteopontin serves as a ligand for CD275, which regulates immune responses by activated T cells [30]. Alternative splicing of osteopontin generates three isoforms: osteopontin-a (the full-length isoform), osteopontin-b (lacking exon 5), and osteopontin-c (lacking exon 4) [31]. Alternative translation of osteopontin produces two isoforms: a cell-secreted full-length and a shortened intracellular protein lacking the N-terminal signal sequence [31]. In addition, osteopontin undergoes numerous posttranslational modifications, such as serine/threonine phosphorylation, sulfation, O-glycosylation, glutamination, and proteolytic processing, which can subsequently determine the functional variability of osteopontin [32]. To date, thrombin, matrix metalloproteinases (MMPs), caspase-8/3, plasmin, cathepsin D, and enterokinase have been identified as proteases that cleave osteopontin at different sites, resulting in the formation of several fragments of different sizes and with variable functions [33]. Taken together, the ultimate roles and functions of osteopontin are impacted by multiple factors, spanning from gene transcription to protein translation, posttranslational modification, and proteasomal processing of the final protein product.

Osteopontin-mediated cellular signaling pathways are well explored in cancer diseases. Thus, interaction of osteopontin with cell surface receptors, integrins, and/or CD44 activates JNK, Ras/Raf/MEK/ERK, PI3K/AKT, JAK/STAT, NF-κB, and TIAM1/Rac1 signaling pathways, leading to enhancement of various malignant properties of cancer cells [34]. Unfortunately, the role of signaling pathways activated by osteopontin in pulmonary hypertension has been poorly studied.

## 3. Osteopontin as a Biomarker of Pulmonary Hypertension and Right Ventricular Failure

Circulating biomarkers are of significant importance in clinical medicine as they can be utilized for diagnostic, prognostic, or therapeutic response monitoring purposes across a broad range of conditions, including PH [35,36]. Osteopontin, a secreted circulating protein, serves as a valuable biomarker due to its accessibility through non-invasive methods such as peripheral blood sampling, which allows frequent and repeated measurements during the course of disease or treatment implementation. Circulating osteopontin has been established as a valuable biomarker of disease severity and adverse outcomes in patients with various cardiovascular conditions including heart failure [37,38,39]. 

PH patients with different etiologies, including IPAH [40,41], PAH associated with congenital heart diseases (CHD-PAH) [42], PAH associated with connective tissue diseases (CTD-PAH) [43] and PH associated with COPD [44], display increased osteopontin expression in the pulmonary vasculature and lung tissue. Osteopontin was found to be one of the top five overregulated genes in the explanted lung tissues of patients with PH of various etiologies, and its expression was directly correlated with the severity of the hemodynamic conditions [41]. Another study demonstrated that osteopontin was one of the nine hub genes in the genome transcriptome datasets evaluated in the lung tissues of PAH patients [45]. In contrast, a recent study demonstrated decreased osteopontin expression in the lung tissue of PAH patients [46]. 

Elevated circulating osteopontin levels were reported in patients with various forms of PH, including IPAH [47], CHD-PAH [42], CTD-PAH [43], and CTEPH [48]. Circulating osteopontin levels were associated with PH development in CTD patients [43] and CHD patients [42]. Increased osteopontin levels were also reported in other cardiac conditions complicated with PH and RV failure, such as dilated cardiomyopathy (DCM) [49]. In a parallel comparison study, it was found that the levels of circulating osteopontin in PH patients increased to a similar extent as they did in patients with DCM and left ventricular hypertrophy compared to healthy controls [47]. Several studies demonstrated a correlation of circulating osteopontin levels with a number of hemodynamic parameters such as mPAP [42], pulmonary artery dispensability index [50], right atrial pressure [51], cardiac index [42], and total pulmonary vascular resistance [42] in PH patients. Similarly, correlation of osteopontin levels with pulmonary hemodynamics and several RV remodeling parameters was demonstrated in patients with DCM [49].

An increase in circulating osteopontin was associated with development of Eisenmenger syndrome in CHD-PAH patients [42]. In PAH patients, baseline circulating osteopontin levels predicted survival [51,52,53] and increased with worsening of the disease condition as assessed by six-minute walking distance (6MWD) [50,51] and New York Heart Association Functional Classification (NYHA-FC) [51,52]. 

Patients with CTEPH had higher osteopontin plasma concentrations compared to patients with pulmonary embolism (PE) [48]. Findings of a higher risk for CTEPH development during follow-up in patients with lower osteopontin levels at diagnosis of acute PE suggest that osteopontin might play an important role in thrombus resolution and thus in the development of CTEPH after PE [48]. Interestingly, pulmonary endarterectomy in CTEPH patients was associated with a further elevation of circulating osteopontin [48]. 

Studies have demonstrated that circulating osteopontin levels predict RV dysfunction and remodeling in PAH patients [50,54]. PH patients with maladaptive RV remodeling displayed higher levels of circulating osteopontin compared to those with adapted RV and left heart failure patients with DCM and left ventricular hypertrophy [47]. Interestingly, circulating osteopontin levels were significantly higher in CTEPH patients with maladaptive RV remodeling compared to those with adapted RV and to IPAH patients with both adapted and maladapted RV [47]. 

**Figure 1:**
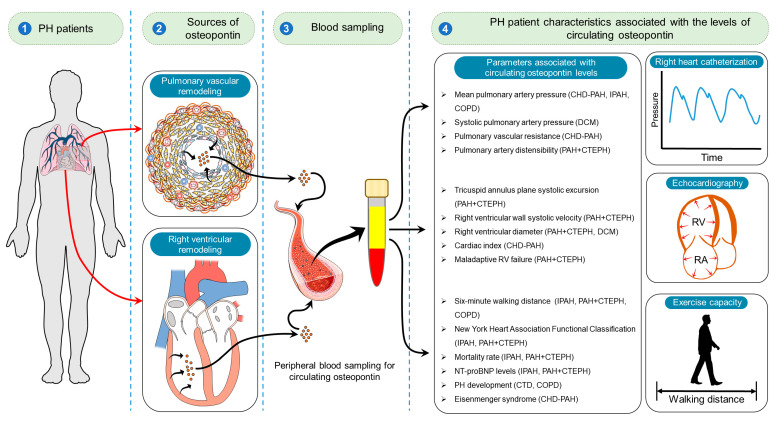
Clinical role of osteopontin. In patients with pulmonary hypertension (PH) of various etiologies (**1**), changes to the pulmonary vessels and the right ventricle (RV) (**2**) cause enhanced osteopontin release into the bloodstream. Circulating osteopontin can be measured in plasma or serum samples (**3**). Numerous studies (**4**) have found that osteopontin levels are increased in PH patients, and these elevated levels are linked to invasive hemodynamic alterations, changes in the functional and structural parameters of the RV, and adverse outcomes. CHD-PAH, pulmonary artery hypertension associated with congenital heart disease; IPAH, idiopathic pulmonary artery hypertension; COPD, chronic obstructive pulmonary disease; DCM, dilated cardiomyopathy; CTD, connective tissue disease; CTEPH, chronic thromboembolic pulmonary hypertension; RA, right atrium.

Taken together, the accumulated evidence suggests that osteopontin might serve as a valuable biomarker for detection of perturbations in pulmonary hemodynamics, exacerbation of RV dysfunction, and prediction of adverse outcomes in patients with various forms of PH (Table 1 and Figure 1). 

Although the level of osteopontin in the circulation is elevated in PH patients, the specific origin of this increase is still unknown. Several lines of evidence point to the pulmonary vasculature and heart as possible sources of circulating osteopontin. Osteopontin release from the heart into the coronary circulation in proportion to the left ventricular (LV) systolic function and volumes was revealed in patients with a previous anterior wall myocardial infarction [55]. Plasma osteopontin levels decreased significantly in heart failure patients following heart transplantation [38]. However, it is hypothesized that the elevated osteopontin may come from both the RV myocardium and the pulmonary vasculature since both of these compartments are impacted in PH [52]. Based on the studies of LV failure, in which the myocardium is considered the primary source of circulating osteopontin, it can be expected that both the RV and pulmonary vasculature may contribute to the rise in the circulating osteopontin in PH. Indeed, lung contribution to circulating osteopontin was clearly demonstrated previously by measuring the transpulmonary osteopontin gradient in heart failure patients [56] and there is clinical and experimental evidence of increased osteopontin expression in the lungs [41,57] as well as in the right ventricle [57]. The possibility that other organs contribute to elevated circulating osteopontin levels, at least in advanced disease stages with multiple organ dysfunction [56], needs further evaluation. However, the relative contribution of each of these compartments at various stages of the disease needs to be established. Studies that specifically examine the osteopontin levels in the circulation, RV and LV myocardium, and pulmonary vasculature, as well as the relationship between osteopontin and disease severity, will be important in shedding light on the origin of osteopontin in this condition. Identification of the source of elevated osteopontin in PH and understanding the underlying mechanisms may help in development of new diagnostic and therapeutic strategies for patients with this debilitating condition. 

## 4. Osteopontin in Pulmonary Vascular Cells

### 4.1. Osteopontin in Pulmonary Artery Endothelial Cells

Osteopontin plays a crucial role in both the physiology and pathophysiology of endothelial cells in the systemic vasculature. It serves as a vital mediator in the intricate physiological and pathological processes that govern endothelial cells in the pathogenesis of several cardiovascular diseases. Various factors, including aldosterone [58], vascular endothelial cell growth factor (VEGF) [59], and hypoxia [60], regulate osteopontin expression in endothelial cells, and vice versa, it regulates various functions of endothelial cells. Osteopontin stimulates endothelial cell differentiation and angiogenesis via its SVVYGLR fragment [61]. It promotes angiogenesis by mediating endothelial cell attachment to the ECM [62] and synthesis of angiogenic factors by endothelial cells [63]. Osteopontin enhances VEGF expression through AKT and ERK signaling pathways to induce endothelial cell motility, proliferation, and tube formation via the αvβ3-integrin receptor [64]. In turn, VEGF augments expression of osteopontin and αvβ3-integrin in endothelial cells and stimulates integrin-dependent endothelial cell migration [59]. A conserved sequence comprising nine amino acids (RSKSKKFRR) located at the thrombin cleavage site of osteopontin promotes endothelial cell migration, proliferation, and tube formation in vitro [65]. The crucial function of osteopontin in neovascularization is further corroborated by the findings of the compromised angiogenic capacity of endothelial cells derived from osteopontin knockout mice, which is partially restored by exogenous administration of osteopontin [66]. Besides angiogenesis, osteopontin influences several other endothelial cell functions. It increases vascular permeability by downregulating expression of the tight junction proteins ZO-1 and claudin-5 in endothelial cells [67], induces endothelial-to-mesenchymal transition via CD44 receptor in response to shear stress [68] and regulates endothelial cell apoptosis [69]. 

The precise role of osteopontin in the functional regulation of PAECs and its interplay in the pathogenesis of pulmonary vascular remodeling still remains largely unknown. It can be hypothesized that elevated circulating osteopontin levels in PH patients may exert effects on PAECs similar to those on endothelial cells in systemic vasculature, potentially leading to alterations in endothelial cell physiology leading to subsequent pathological pulmonary vascular remodeling. This is due to the established fact that endothelial cell dysfunction plays a crucial role in the pathogenesis of PH, particularly in the early stages of the disease [70]. However, the precise mechanisms by which osteopontin affects PAEC physiology and its potential contribution to the development of PH remains a subject of future research. 

### 4.2. Osteopontin in Pulmonary Artery Smooth Muscle Cells

Osteopontin critically influences physiology of systemic vascular smooth muscle cells (SMCs) through modulation of a myriad of cellular processes, including cell proliferation [71], apoptosis [72], migration [73,74], and neointima formation [75]. Various factors, including hypoxia [76], platelet-derived growth factor (PDGF) [77], hyperglycemia [78], mechanical stress [79], aldosterone [80], and reactive oxygen species (ROS) [81], regulate osteopontin expression in SMCs. In vitro studies revealed that the cell proliferation rate of SMCs is directly related to osteopontin expression levels in these cells [82], suggesting a direct role of osteopontin in mediating cell proliferation. Autocrine expression of osteopontin contributed to PDGF-induced SMC migration [83] and regulated adhesion of SMCs to the ECM [84] and production of MMPs [85]. Osteopontin increased ECM synthesis in SMCs via activating the p38 MAPK signaling pathway [86]. Production of osteopontin in SMCs was inhibited by a cyclic guanosine monophosphate (cGMP)-dependent protein kinase, an important mediator of nitric oxide (NO) and cGMP signaling [87]. Pharmacological agents enhancing NO-cGMP signaling might thus potentially attenuate osteopontin expression. 

Available data suggest that osteopontin regulates various cellular functions of PASMCs, similar to the systemic vascular SMCs. Osteopontin was found to be as one of the most highly overregulated matricellular proteins in senescent PASMCs [40]. Its expression in these cells was associated with an augmented cell migration and proliferation [40], which were markedly reduced in the presence of neutralizing anti-osteopontin antibodies [40]. 

Multiple factors with known pathological roles in the pulmonary vascular remodeling, including acidic fibroblast growth factor [88], angiotensin-II [88], transforming growth factor-β [89], PDGF-BB [90], hypoxia [91,92], sphingosine-1-phosphate [93], and mechanical stretch [42], induce osteopontin expression in PASMCs through various signaling pathways. Acidic fibroblast growth factor induced osteopontin expression in PASMCs via activating Ras/JNK and Ras/MEK1/2 signaling pathways [88], while hypoxia did so via ERK and p38MAPK signaling pathways [92] and mechanical stretch via AKT-ERK signaling pathways [42]. In turn, osteopontin modulates various functions of PASMCs. In particular, it promoted PASMC proliferation and migration in a dose-dependent manner via αVβ3-integrin mediated AKT and ERK1/2 signaling pathways [42]. Furthermore, activation of the calcineurin/NFATc3 signaling pathway by sphingosine-1-phosphate upregulated osteopontin expression and stimulated PASMCs proliferation, which was suppressed by the activation of the proliferator-activated receptor gamma (PPAR-γ) [93].

### 4.3. Osteopontin in Pulmonary Artery Adventitial Fibroblasts

Recent research demonstrated the significance of vascular adventitia and adventitial fibroblasts in maintaining vascular homeostasis of both systemic [94] and pulmonary vasculature [95]. The adventitial fibroblasts are involved in the regulation of various cellular processes, including ECM remodeling, inflammation, and angiogenesis, which are critical for maintaining the structural and functional integrity of the vasculature [94,95]. In the systemic vasculature, osteopontin plays a significant role in SMC-mediated regulation of adventitial fibroblast functions [96]. Integrin β3 was found to be the responsible receptor for promoting osteopontin-mediated adventitial fibroblast migration [97]. The role of osteopontin in the physiology of pulmonary vascular adventitial fibroblasts was studied primarily in the bovine model of PH. Adventitial fibroblasts isolated from calves with severe hypoxia-induced PH exhibited high proliferative, migratory, and pro-invasive capabilities. These functional alterations of adventitial fibroblasts were mediated via activated ERK1/2 and AKT signaling pathways that correlated with high osteopontin expression [28]. Inhibition of osteopontin expression with a specific small interfering RNA or neutralizing antibodies led to an attenuation of proliferative, migratory, and invasive capabilities of adventitial fibroblasts [28]. 

### 4.4. Osteopontin in Pulmonary Vascular Macrophages

The literature pertaining to the specific functions of osteopontin in the functional dynamics of macrophages and their interactions in the pathogenesis of PH is scarce. In hypoxia, osteopontin can act as an inflammatory cytokine, thereby promoting the chemotaxis of various inflammatory cells and modulating the inflammatory milieu in the affected tissue. Although circulating monocytes do not express osteopontin, it is among the most highly expressed genes in activated macrophages [98]. Additionally, it serves as a powerful chemotactic stimulus for these immune cells [99]. Osteopontin regulates key functions of macrophages, including migration, survival, phagocytosis, and pro-inflammatory cytokine production [100,101]. These effects are mainly mediated via interaction with α4/α9-integrins by its SLAYGLR domain [102]. 

The potent pro-inflammatory properties of osteopontin are further augmented following its cleavage by thrombin, which leads to the generation of an N-terminal fragment containing two integrin-binding domains, namely the RGD and SVVYGLR motifs [103]. Osteopontin can contribute to pulmonary vascular remodeling through facilitating attraction and retention of macrophages and T lymphocytes in areas of inflammation within pulmonary vessels [104]. A single-cell RNA sequencing demonstrated that enhanced osteopontin expression in lung tissues from patients with systemic sclerosis was enriched in macrophages [98], suggesting that osteopontin signaling in these cells might contribute to the pulmonary vascular remodeling in PAH associated with systemic sclerosis. 

Thus, available data suggest that osteopontin might contribute to the chemotaxis of leukocytes and other inflammatory cells to the remodeled pulmonary vasculature and thereby be involved in the pathogenesis of the disease. However, the intricacies of osteopontin function within pulmonary vascular macrophages and other immunocompetent cells and their contribution to pulmonary vascular remodeling remain enigmatic, warranting future investigation.

### 4.5. Osteopontin in Intercellular Communications of Vascular Cells in Pulmonary Vascular Remodeling

**Figure 2:**
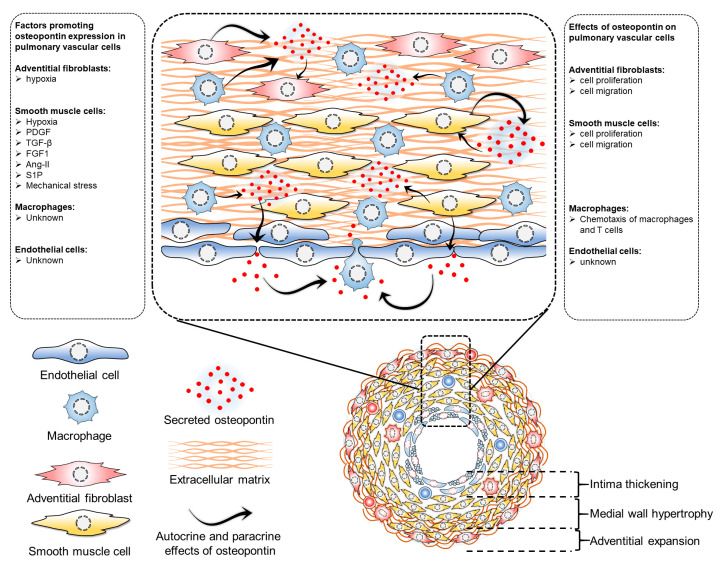
Regulation of osteopontin expression and effects of osteopontin in pulmonary vascular cells. Several factors regulate osteopontin expression in pulmonary vascular cells. In adventitial fibroblasts, hypoxia induces osteopontin expression, which has implications in cell proliferation and migration. In pulmonary artery smooth muscle cells, many factors regulate osteopontin expression, including hypoxia, PDGF (platelet-derived growth factor), TGF-beta (transforming growth factor beta), FGF1 (fibroblast growth factor 1), Ang-II (angiotensin II), S1P (sphingosine 1-phosphate), and mechanical stress, all of which also regulate cell proliferation and migration. However, the exact factors regulating osteopontin in pulmonary vascular endothelial cells and macrophages have not been identified. While osteopontin regulates macrophage chemotaxis, its role in endothelial cells remains unknown.

Elevated circulating osteopontin levels in PH can be attributed to its enhanced synthesis by pulmonary vascular cells. The intricate regulation of osteopontin expression by various factors including hypoxia, growth factors, mechanical stress, and inflammation in these cells highlights its complexity. Direct effects of osteopontin on pulmonary vascular cells through both paracrine and autocrine mechanisms suggest its potential role in regulating cellular proliferation, migration, and other functions in these cells, thereby potentially serving as a key mediator in intercellular communication within the pulmonary vasculature (Figure 2). These effects of osteopontin on pulmonary vascular cells adversely affect the disease and may determine the impact of circulating osteopontin on the status and outcome of PAH patients. However, the intricacy of these interactions, particularly concerning the extent of their contribution and hierarchical involvement in the pathological processes of pulmonary vascular remodeling, presents a significant challenge in precisely defining the cell-specific role of osteopontin in pulmonary vascular cells.

## 5. Osteopontin in Animal Models of Pulmonary Hypertension

Utilization of experimental models for the study of PH has been a crucial methodology for understanding the underlying mechanisms and causes of this condition, as well as for the preclinical advancement of the currently approved therapeutic approaches. The established models of PH continue to play a vital role in facilitating further research and exploration of potential innovative treatment options for the management of PH [105,106]. 

Increased lung expression of osteopontin was demonstrated in several rodent PH models, including hypoxia exposure in mice [107,108] and rats [46,88,104] and monocrotaline injection in rats [109,110]. These studies suggested that osteopontin release from remodeled pulmonary vessels could account for elevated circulating osteopontin levels [110]. Examination of lung tissues revealed generation of osteopontin in the remodeled vessels by pulmonary fibroblasts [28,104] and PASMCs [110,111]. In addition, osteopontin levels may reflect the extent of pulmonary vascular remodeling. For example, atrial natriuretic peptide null mice exhibit augmented lung osteopontin expression, which is associated with an intensive pulmonary vascular remodeling [107], while mice with mutated transforming growth factor-beta receptor type 2 display attenuated pulmonary vascular remodeling and blunted osteopontin expression in response to hypoxia exposure [108]. In addition, attenuation of lung osteopontin expression may indicate beneficial effects of a particular drug tested in animal models of PH. For example, pioglitazone, an activator of PPAR-γ, improved pulmonary vascular and RV remodeling in monocrotaline rats along with attenuating osteopontin expression in the lung [109]. Similarly, the beneficial effects of calcium-sensing receptor antagonist NPS2390 [111] and serotonin transporter inhibitor fluoxetine [110] in animal PH models was associated with decreased lung tissue osteopontin expression along with improvement in pulmonary hemodynamics. Collectively, the pulmonary osteopontin expression level corresponds to the extent of pulmonary vascular remodeling in multiple animal PH models. These studies demonstrate that osteopontin can be utilized as a means of monitoring the impact of potential pharmacological agents in rodent PH models. 

Several experimental models, including aging-associated and high-flow-induced PH, have nicely demonstrated the pivotal role of osteopontin in the pathogenesis of pulmonary vascular remodeling. Mice lacking osteopontin were protected from age-induced pulmonary vascular remodeling and displayed attenuated pulmonary vascular remodeling in response to hypoxia exposure [40]. In a rat model of shunt-induced PH, pulmonary expression of osteopontin and its two main receptors αVβ3-integrin and CD44 increased along with the disease worsening [42]. Interestingly, application of an αvβ3-integrin antagonist in surgically corrected shunt rats accelerated recovery from the pulmonary vascular remodeling and RV failure [42]. 

**Figure 3:**
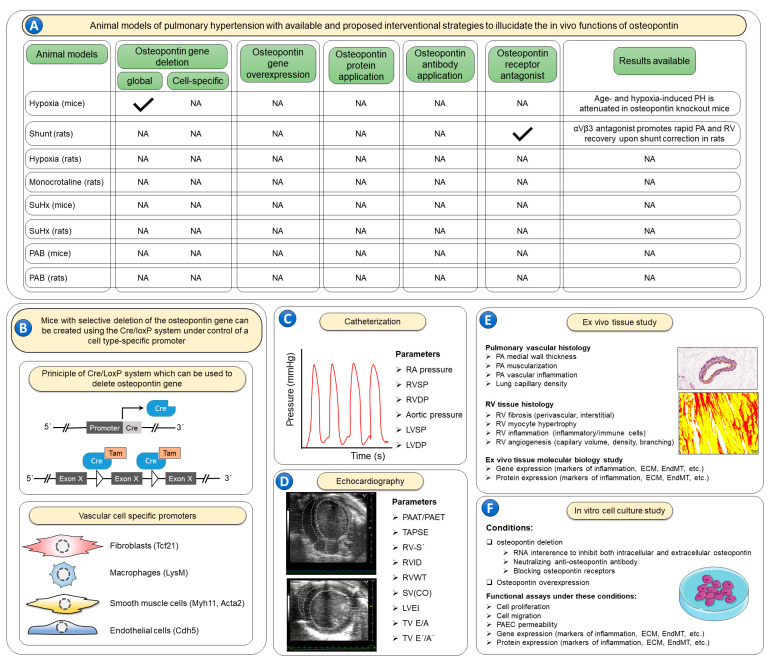
Possible implications of experimental pulmonary hypertension models in future studies. (**A**) Among many available mouse and rat pulmonary hypertension models, listed here (hypoxia, shunt, monocrotaline, sugen plus hypoxia (SuHx), pulmonary artery banding (PAB)), osteopontin-targeted studies were performed only in hypoxia-exposed global osteopontin knockout mice and in a rat model of shunt-induced pulmonary hypertension using osteopontin receptor (αVβ3) antagonist. Osteopontin-oriented in vivo studies in other pulmonary hypertension models are still missing. There have been no studies with cell-specific osteopontin deletion or overexpression in rodent pulmonary hypertension models. 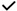
—indicates that animal models with the corresponding osteopontin manipulations are available; NA—studies with the corresponding animal models and osteopontin manipulations are not available. (**B**) No experimental studies have evaluated effects of recombinant osteopontin application or osteopontin-neutralizing antibodies. In order to elucidate the cell specific roles of osteopontin, the Cre/LoxP system can be utilized using cell-specific promoter systems. Further, to better characterize the role of osteopontin in such models it is recommended to use invasive catheterization (**C**) to measure right atrial (RA) pressure, right ventricular (RV) systolic pressure (RVSP), RV diastolic pressure (RVDP), aortic pressure, left ventricular (LV) systolic pressure (LVSP) and LV diastolic pressure (LVDP). Echocardiographic imaging (**D**) of the heart is also warranted to inform additional characteristics of the RV including both systolic and diastolic functions, including the following parameters: the ratio of pulmonary artery acceleration time to pulmonary artery ejection time (PAAT/PAET), tricuspid annulus systolic excursion (TAPSE), RV annulus systolic velocity (RV-S´), RV internal diameter (RVID), RV wall thickness (RVWT), stroke volume (SV), cardiac output (CO), LV eccentricity index (LVEI), tricuspid valve inflow velocities (TV E/A), and tricuspid annulus lateral velocities (TV E´/A´). Following the terminal catheterization and echocardiography assessments, lung and heart tissues (**E**) can be evaluated ex vivo for pulmonary artery (PA) wall thickness, muscularization and inflammation, as well as lung capillary density. RV tissue can be assessed for RV fibrosis, cardiomyocyte hypertrophy, inflammation, and angiogenesis. Furthermore, lung and RV tissues can be studied for the expression of genes and proteins involved in various pathological processes including inflammation, extracellular matrix (ECM) synthesis and endothelial-to-mesenchymal transition (EndMT). Finally, the exact cellular roles of osteopontin can be studied in vitro (**F**) using cell culture techniques under both osteopontin loss- and gain-of-function conditions to assess cell proliferation, migration, and apoptosis. Employing such strategies in rodent pulmonary hypertension models, and ex vivo tissue and in vitro cell culture experiments may be necessary to fully characterize the role of osteopontin in pulmonary hypertension.

There is still a lack of studies investigating the specific actions of osteopontin within individual pulmonary vascular cells using genetically modified mice lacking or overexpressing osteopontin in the experimental PH models (Figure 3). Using cell-specific conditional knockout animals in addition to global osteopontin knockout animals may add more value to exploring cell-specific roles of osteopontin in PH. In addition, conditional gene knockouts allow overcoming some of the limitations of conventional knockouts, such as early embryonic lethality, compensation by another gene product during development, and alterations of other organ systems. Another key advantage of conditional gene knockouts is that by knocking out the gene of interest in specific tissues or cell types, it is possible to assign a phenotype to a particular cell type. Further, gene inactivation at a specific time point allows determining the role of the protein in the initiation vs. progression of the disease. 

Effects of osteopontin in severe PH models, such as SuHx or monocrotaline injection, are yet to be investigated. Moreover, there are no experimental studies investigating the effects of the administration of recombinant osteopontin to increase its levels or the use of osteopontin-neutralizing antibodies to decrease its levels in circulation in PH models (Figure 3). Cumulatively, the biological significance of osteopontin in animal PH models requires further investigation in order to fully comprehend its role in the development and progression of pulmonary vascular remodeling. This information will provide a conclusive answer regarding the potential benefits of manipulating osteopontin levels for managing PH patients.

## 6. Osteopontin in Right Ventricular Remodeling

Numerous investigations utilizing both in vivo animal models and human clinical studies have demonstrated a crucial role for osteopontin in the pathogenesis of LV failure of various etiologies [112]. However, there remains a paucity of data pertaining to the specific role of osteopontin in RV dysfunction and failure. Accumulating evidence suggests that osteopontin may play a significant role in RV pathologies. Similar to the pulmonary vascular remodeling, remodeled RV may represent an important source of circulating osteopontin. A correlation between the plasma concentrations of osteopontin and the expression levels within the hypertrophied RV myocardium was demonstrated in monocrotaline and SuHx rat PH models [113,114]. Recent transcriptomic analysis of RV tissues from monocrotaline and SuHx rats revealed that osteopontin was one of the top nine overregulated genes [115], suggesting that pressure overload induces osteopontin expression in the RV.

The proposition that osteopontin may negatively impact the RV is partially predicated upon the findings of various studies that reported a reduction in osteopontin expression following treatment with pharmacological agents that improve pulmonary hemodynamics and RV function, such as PPAR-γ activator pioglitazone [109] and estrogen receptor-β agonist 17β-estradiol [113]. While it is possible that this decrease in osteopontin expression is simply a reflection of the reduced afterload, which subsequently mitigates the stress exerted upon the RV wall, it cannot be entirely ruled out that these agents may exert a direct impact on the RV and thereby attenuate osteopontin expression. Therefore, it is crucial to perform further investigations utilizing the afterload-independent experimental models of RV failure, in which the concentrations of osteopontin are either enhanced via exogenous administration or decreased through the utilization of genetically modified knockout organisms or neutralizing antibodies, in order to either confirm or refute such hypotheses. 

## 7. Osteopontin as a Treatment Target in Pulmonary Hypertension

Despite the wealth of data implicating osteopontin in the pathogenesis of PH, studies employing specific therapeutic approaches directly targeting osteopontin, such as osteopontin-neutralizing antibodies or osteopontin aptamers in preclinical PH models, are still lacking. Studies based on the cancer pathologies demonstrated a therapeutic potential of various approaches to suppress osteopontin using aptamers, antibodies, and small molecular as well as the miRNA-based medications [116]. Among these many options, aptamers have emerged as one of the promising strategies to target osteopontin more specifically and precisely. Osteopontin aptamers effectively block osteopontin function in vitro [117] and in vivo [118] and thereby provide therapeutic benefits. Similarly, osteopontin-neutralizing antibodies suppressed osteopontin in various heart failure models [119,120]. Blocking osteopontin receptors represents another interesting approach to inhibit its effects. Application of an αVβ3-integrin antagonist accelerated recovery from the pulmonary vascular remodeling and RV failure in surgically corrected shunt rats [42]. Taken together, despite the accumulating evidence implicating osteopontin in the pathogenesis of PH, the field has yet to fully explore the potential therapeutic utility of osteopontin inhibition through various modalities, including but not limited to: RNA interference utilizing small interfering RNAs, short hairpin RNAs, aptamers, monoclonal antibodies, and small molecular inhibitors. Further investigations in animal models are imperative in order to evaluate the potential benefits of osteopontin inhibition in the management of PH. 

## 8. Future Experimental Perspectives

Available data suggest that osteopontin plays a pivotal role in pulmonary vascular remodeling by regulating various functions of pulmonary vascular cells. However, they do not provide a definitive clue on the precise roles of osteopontin in PH. Consequently, several issues need to be addressed in future studies. First, although enhanced osteopontin expression in lung and heart tissues was demonstrated in several animal PH models, the specific roles of osteopontin were studied in only a few of them. These models include hypoxia-exposed global osteopontin knockout mice [40] and a rat model of shunt-induced PH that used an osteopontin receptor (αVβ3) antagonist [42]. In both models, blocking osteopontin was associated with improved pulmonary hemodynamics and pulmonary vascular remodeling. Nevertheless, there is still a lack of experimental studies examining the in vivo role of osteopontin in other PH models such as monocrotaline injection, SuHx, and pulmonary artery banding models. Moreover, no studies examined effects of cell-specific osteopontin deletion or overexpression, recombinant osteopontin application, or osteopontin-neutralizing antibodies in rodent PH models. Utilization of the Cre/LoxP system with cell-specific promoters could provide information on the cell-specific roles of osteopontin in vivo, ultimately determining the main cell type in the pulmonary vasculature involved in the development of PH. Such precise cell-specific strategies may have implications for the development of successful osteopontin-specific therapeutics.

It is crucial to employ in future studies state-of-the-art techniques, including magnetic resonance imaging, echocardiography, and catheterization, to better characterize the effects of osteopontin loss- and gain-of-function strategies on pulmonary hemodynamics and cardiac function/structure. Then, lung and RV tissues can be subjected to multi-omics analysis to give insights into the upstream and downstream signaling pathways underlying osteopontin-mediated pathological processes in pulmonary vascular remodeling. PASMCs are the most frequently used pulmonary vascular cell type to study the cellular roles of osteopontin in PH. The exact regulators of osteopontin and its functional effects in other cell types in the pulmonary vasculature remain unclear. Thus, the exact cellular roles of osteopontin require further study using in vitro cell culture techniques under both osteopontin loss- and gain-of-function conditions (Figure 3).

Osteopontin may be deemed a viable therapeutic target for the treatment of PH. However, prior to the advancement of osteopontin-directed therapeutics in PH, the precise functions of full-length osteopontin and its various fragments and their implications in the pathogenesis of PH must be carefully elucidated. This will allow identification of those osteopontin fragments that should be specifically targeted. Such a precise, accurate manipulation of the osteopontin system will contribute to the amelioration of the pulmonary vascular and RV remodeling and affect positively the natural trajectory of PH, ultimately resulting in improved outcomes for PH.

## 9. Summary

The purpose of this review was to illuminate the crucial role of osteopontin and related signaling pathways in the pathogenesis of pulmonary vascular remodeling. Strong evidence has accumulated for the critical role of osteopontin in pulmonary vascular remodeling. Osteopontin is a key component in the regulation of complex signaling events that drive aberrant cellular functions in the pulmonary vasculature. Its direct actions on pulmonary vascular cells, such as augmented cellular proliferation, migration, and ECM synthesis, are considered salient mechanisms underlying osteopontin-mediated pathological pulmonary vascular remodeling. Its pro-inflammatory actions promote infiltration of inflammatory cells into the pulmonary vascular wall, thereby further negatively affecting the pulmonary vascular remodeling. These pivotal cellular contributions of osteopontin in PH are further corroborated by studies utilizing rodent PH models, in which osteopontin deficiency resulted in the amelioration of PH phenotype in mice exposed to hypoxia or promoted pulmonary vascular reverse remodeling in a shunt model of PH. In PAH patients, osteopontin has become an established marker of altered pulmonary hemodynamics and adverse outcomes. A strong relationship was demonstrated between its circulating levels and the extent of alterations in pulmonary hemodynamics and RV structure and function, and functional capacity in PH patients. Owing to its role in the pathogenesis of pulmonary vascular remodeling, osteopontin may represent a viable target for the treatment of PAH. While specific strategies such as silencing osteopontin expression or inhibiting its activity have yet to be utilized in rodent PH models, such approaches may provide a valuable insight into the potential utility of osteopontin suppression in the treatment of this condition. Further research is necessary to fully understand the mechanisms by which osteopontin regulates adverse pulmonary vascular remodeling and to address numerous unanswered questions in this field.

## Figures and Tables

**Table 1:** Summary of clinical studies evaluating circulating osteopontin levels in patients with various forms of pulmonary hypertension. Abbreviations: PA, pulmonary artery; PH, pulmonary hypertension; IPAH, idiopathic pulmonary arterial hypertension; RV, right ventricle; NYHA-FC, New York Heart Association functional classification; WHO-FC, World Health Organization functional class; RVEDD, RV end-diastolic diameter; TAPSE, tricuspid annulus plane systolic excursion; RV-S´, tricuspid annulus systolic velocity; RVD1, RV basal diameter; RVD2, RV mid-diameter; NT-proBNP, N-terminal pro-b-type natriuretic peptide; 6MWD, six-minute walking distance; PAP, pulmonary artery pressure; mPAP, mean PAP; sPAP, systolic PAP; RVD, right ventricular dysfunction; PA, pulmonary artery; CAD, coronary artery disease; DCM, dilated cardiomyopathy; CTD, connective tissue disease; CHD, congenital heart disease, COPD, chronic obstructive pulmonary disease; MRI, magnetic resonance imaging; CI, cardiac index; TPVR, total pulmonary vascular resistance; CTEPH, chronic thromboembolic pulmonary hypertension; LVH, left ventricular hypertrophy.

Subjects	Main Findings	Studies
IPAH (n = 35)	Circulating osteopontin levels correlated with WHO-FC and 6MWD. A cut-off of osteopontin (53.4 ng/mL) predicted significant differences in survival at 4.0 ± 2.2-year follow-up.	[53]
IPAH (n = 95) (retrospective cohort (n = 70), prospective cohort (n = 25), control (n = 40)	In both retrospective and prospective cohorts, circulating osteopontin levels correlated with mPAP and NT-BNP. In the retrospective cohort, osteopontin levels also correlated with age, 6MWD, and NYHA class. Multivariate Cox analysis demonstrated that baseline osteopontin levels were independent predictors of mortality.	[51]
PH (n = 71), control (n = 40)	Patients with advanced right heart failure revealed higher levels of circulating osteopontin compared to less symptomatic ones (NYHA III-IV vs. NYHA I-II). Osteopontin was a strong independent predictor of all-cause mortality within 24 months of follow-up.	[52]
PH (PAH + CTEPH) (n = 71)	Circulating osteopontin levels correlated with RVEDD, TAPSE, and RV-S´. Patients with RV dysfunction had higher levels of osteopontin compared to those without RV dysfunction (956 ng/mL vs. 628 ng/mL). ROC analysis revealed that an osteopontin concentration of 694.2 ng⁄mL detects RV dilatation.	[54]
PH (PAH + CTEPH) (n = 62), control (n = 12)	Circulating osteopontin levels in PH patients were elevated compared to those in healthy control subjects. Circulating osteopontin levels predicted decreased 6MWD. Osteopontin levels were associated with NT-proBNP, RVEDD (echo), RVD (MRI), and PA distensibility index.	[50]
CAD-COPD (n = 131)	Circulating osteopontin levels correlated with mPAP and 6MWD. Osteopontin levels > 43 ng/mL were a statistically significant predictor of PH in patients with CAD-COPD.	[44]
CHD (n = 22), CHD-PAH (n = 25), control (n = 24)	Circulating osteopontin levels increase with the development of PAH and Eisenmenger syndrome. Circulating osteopontin levels correlated with mPAP, CI, and TPVR.	[42]
DCM (n = 70) (DCM without RVD (n = 15) and with RVD (n = 55))	Circulating osteopontin levels in DCM patients correlated with RVD1, RVD2, and sPAP.	[49]
PH (n = 62), DCM (n = 34), LVH (LVH; n = 47), control (n = 38)	Circulating osteopontin levels were higher in PH, DCM, and LVH patients compared to those in the controls. Osteopontin concentrations in PH patients with maladaptive RV were significantly higher than in those with adaptive RV of CTEPH origin. In PH patients, osteopontin levels were correlated with TAPSE/sPAP, RVEDD, mPAP, PVR, NT-pro-BNP, NYHA-FC.	[47]
CTD (n = 113)	CTD-PAH patients showed significantly higher circulating osteopontin levels than patients with CTD alone. Osteopontin levels were independently associated with PAH diagnosis.	[43]

## Data Availability

Not applicable.
